# The Effect of Missing Item Data on the Relative Predictive Accuracy of Intimate Partner Violence Risk Assessment Tools

**DOI:** 10.1177/10731911251386519

**Published:** 2025-11-12

**Authors:** Bronwen Perley-Robertson, Anna T. Pham, N. Zoe Hilton

**Affiliations:** 1 Department of Psychology, Carleton University, Ottawa, Ontario, Canada; 2 Sexual Misconduct Support and Resource Centre, Department of National Defence, Ottawa, Ontario, Canada; 3 Department of Psychiatry, University of Toronto, Ontario, Canada; 4 Waypoint Research Instutite, Waypoint Centre for Mental Health Care, Penetanguishene, Ontario, Canada

**Keywords:** intimate partner violence, risk assessment, missing data, predictive accuracy, ODARA, SARA

## Abstract

In an innovative simulation study, Perley-Robertson et al. found that two correctional risk assessment tools were robust to missing data, with summation, proration, and multiple imputation producing nearly identical relative predictive validity results. However, the uniform deletion of items across cases may have preserved their risk rankings and, consequently, relative predictive accuracy. We extend this research by applying identical missing data conditions (1%–50% of items deleted in 10% increments) to one third, two thirds, and three thirds of a high-risk intimate partner violence (IPV) sample assessed on the Ontario Domestic Assault Risk Assessment (ODARA) and Spousal Assault Risk Assessment–Version 2 (SARA-V2; *N* = 267). Neither missing data nor the handling method affected relative predictive accuracy, though summation underestimated absolute risk. These findings support proration or multiple imputation when IPV risk scale items are missing within a research sample, and underscore that proration is preferable to summed totals in practice.

Risk assessment tools are widely used in the criminal legal system to inform sentencing and conditional release decisions, prioritize individuals for correctional interventions, and manage risk in the community (Bonta & Andrews, 2007; Viljoen et al., 2025). Instruments designed for evaluating intimate partner violence (IPV) risk have been tested extensively across correctional, policing, treatment, and research contexts (Allard et al., 2024; Perley-Robertson et al., 2025; Svalin & Levander, 2020). However, minimal attention has been paid to the effect of missing data on the predictive accuracy of these tools (Perley-Robertson et al., 2024). This oversight is surprising because missing data are common in both prospectively (e.g., Belfrage et al., 2012; Storey et al., 2014) and retrospectively scored IPV samples (e.g., Jung & Buro, 2017; Radatz & Hilton, 2022).

Recognizing this gap in the broader literature, Perley-Robertson et al. (2024) conducted a simulation study examining how missing data affected the relative predictive accuracy of two widely used risk assessment tools: the Spousal Assault Risk Assessment–Version 2 (SARA-V2) and the STABLE-2007. They tested six different conditions, starting with the mostly complete observed data and progressing through five conditions where they randomly deleted 1% to 50% of scale items in 10% increments. Briefly, missing data were then addressed using three techniques (discussed in more detail below): summation (the sum of available items, hereafter referred to as *mechanical scoring*), proration (the average of available items), and multiple imputation (an advanced technique that fills in missing values through statistical modeling).

Interestingly, neither the amount of missing data nor the technique used to address them affected relative predictive accuracy, but mechanical scoring underestimated absolute risk (average total scores decreased by roughly 50% from the first to last conditions). However, a key limitation of Perley-Robertson and colleagues’ (2024) study was the generation of missing data across the entire sample (e.g., every case had 1%–10% of their scale items deleted in the first missing data condition). The researchers noted that this method applies roughly the same amount of measurement error to all cases, which may have inadvertently preserved their relative risk rankings. This, in turn, could explain why relative predictive accuracy estimates—measured using rank-order statistics—remained stable despite the high level of missing data. The present study addressed this methodological limitation by testing missingness in one third and two thirds of the sample, in comparison with the entire sample.

In addition, Perley-Robertson and colleagues’ (2024) sample was restricted to individuals serving a community sentence, whereas IPV risk assessment tools are commonly used in policing and pretrial contexts. Their SARA-V2 sample also had dual IPV and sexual offending histories, potentially limiting the generalizability of their results. The present study was conducted to address this sample limitation, as well as to extend missing data research to the Ontario Domestic Assault Risk Assessment (ODARA), another IPV risk assessment tool that is widely used (e.g., see Goossens et al., 2024).

## The Context of IPV Risk Assessment

IPV risk assessment tools should aid practitioners’ appraisal of a case, provide a framework for supporting and auditing decisions, and improve the consistency and effectiveness of risk communication across sectors (Kebbell, 2019; Saxton et al., 2022). However, several practical constraints affecting implementation can lead to missing data, which may undermine these goals. For example, practitioners often assess risk under time pressures, such as when safety planning is urgent and pretrial decisions are imminent (Svalin & Levander, 2020). Training limitations may also contribute to assessment problems. In one study, most police officers reported using IPV risk assessment tools, but fewer than half received formal training (Campbell et al., 2018). Other research suggests that police officers may only receive brief training (Belfrage et al., 2012) or receive training months or years before applying the tool (Hilton et al., 2024).

Potentially illustrating these implementation challenges, police and community supervision officers have been found to rate items as missing when evidence indicated they were either present or absent (Belfrage et al., 2012; Maltais, 2025). In Belfrage and colleagues’ study, these omissions were likely due, in part, to time constraints and the brief risk assessment training officers received. Support for this explanation comes from Storey and colleagues (2014), who observed fewer item omissions in their follow-up study on a related tool. They attributed this improvement to officers having gained more experience conducting risk assessments since their original study—experience that also included further training. Regarding time pressures, they noted that supervisors, aware of the many item omissions documented by Belfrage et al., may have encouraged officers to complete more comprehensive assessments.

In addition to these implementation problems, certain information is particularly difficult to obtain in policing contexts. Mental health items are challenging to score because officers typically lack access to perpetrators’ health care histories or may not have the expertise to evaluate mental health status (Hilton et al., 2021; Jung & Buro, 2017). Victim or survivor information may also be unavailable when these individuals cannot be reached, such as in institutional settings where assessments occur well after the offense (Gray et al., 2025; Hilton et al., 2021) or when the victim cannot be interviewed due to severe injury or lethality (Campbell et al., 2009).

### Guidelines for Addressing Missing Data in IPV Risk Assessment

Developers of IPV risk tools recognize these assessment challenges but provide different instructions for handling missing information. The manuals for the SARA-V2 (Kropp, Hart, Webster, & Eaves, 2008) and Brief Spousal Assault Form for the Evaluation of Risk (B-SAFER; Kropp et al., 2010) advise users to omit items when there is insufficient information to score them, and to discuss any resulting limitations in their assessment reports. These omissions do not preclude evaluators from making a final risk judgment. Neither the SARA-V2 nor the B-SAFER manual provides guidelines on how to handle missing items in research, where total scores are often used to evaluate predictive accuracy (Allard et al., 2024; Svalin et al., 2018).

In contrast, the ODARA manual provides a proration table for handling missing information, which allows proration for up to five items if the information needed to score them is unclear or only partially complete (Hilton, 2021). Users are advised to gather as much information as they can before resorting to proration, especially regarding criminal history items. If more than five items cannot be scored, the ODARA’s use is not recommended. These same rules apply to the Domestic Violence Risk Appraisal Guide (DVRAG), which is an algorithm for combining the ODARA with the Psychopathy Checklist-Revised (Hare, 2003), although prorating is more complex due to the DVRAG’s weighted scoring system (Hilton, 2021).

Other tools provide little to no instruction on handling missing information. For example, the Guidelines for Stalking Assessment and Management merely encourages evaluators to gather the required information (Kropp, Hart, & Lyon, 2008). The Idaho Risk Assessment of Dangerousness only instructs users to note when a survivor is unable or unwilling to respond to a question (Idaho Coalition Against Sexual and Domestic Violence, 2021). Missing items are scored as “unknown” in the Domestic Violence Screening Instrument (Williams & Houghton, 2004) and are simply excluded from the total score, though we could not obtain the user manual to see if explicit instructions are provided. The Danger Assessment scoring sheet (Campbell, 2019) does not provide guidelines for handling missing information, and neither does the user manual adapted by Alberta Council of Women’s Shelters (2019). Another tool without missing data guidelines is the Domestic Violence Risk and Needs Assessment (Colorado Department of Public Safety, 2016).

### Application of Missing Data Approaches in IPV Risk Assessment Research

Researchers have implemented a diverse range of missing data handling techniques as a result of the varied guidelines for IPV risk tools. This inconsistency may introduce unknown biases into the literature, making it difficult to compare results across studies. For example, some researchers have used listwise or pairwise deletion, excluding cases with missing data entirely (Jung & Buro, 2017; Nazarewicz et al., 2024) or on an analysis-by-analysis basis (Kropp & Hart, 2000; López-Ossorio et al., 2017). Others have modified scales by excluding items that could not be reliably scored from police records (Jung & Buro, 2017; Radatz & Hilton, 2022). To retain incomplete cases and items, most researchers have employed mechanical scoring (Belfrage et al., 2012; Gray et al., 2025; Jung & Himmen, 2022; Murphy et al., 2003; Storey et al., 2014) or proration (Grann & Wedin, 2002; Gray et al., 2025; Pham et al., 2023; Vølstad et al., 2025).

## Approaches to Missing Data in Correctional Risk Assessment

Given the varying recommendations from IPV risk tool developers and application of diverse missing data handling approaches, it is important to understand the theoretical foundations and practical implications of each. Deletion methods reduce statistical power and can introduce bias into the results if there is systematic missingness (Little & Rubin, 2002). Scale modifications reduce the generalizability of results and may degrade predictive accuracy if the strongest predictors are omitted. As mechanical scoring and proration retain incomplete cases and items, researchers likely view them as better options, hence their widespread use. Yet only one study has compared their performance against each other and to more sophisticated techniques like multiple imputation (Perley-Robertson et al., 2024). The present study extends this work, beginning first with a brief description of each method—mechanical scoring, proration, and multiple imputation—as summarized by Perley-Robertson et al. (2024, pp. 1644–1646).

### Mechanical Scoring

The simplest of these three approaches is to mechanically sum the available items. Although convenient, this method can underestimate risk by effectively assigning zeros to missing items (Downey & King, 1998). Perley-Robertson and colleagues discussed how this method could degrade the predictive accuracy of risk tools by reducing the association between scale scores and recidivism for recidivists who would have scored higher than zero on missing items. Ignoring missing items by summing without correction also redefines the composition of the scale by the level and pattern of missing data. Applying Schafer and Graham’s (2002) logic for proration, this limits the generalizability of results across samples. Perley-Robertson et al. also discussed how this method could degrade the predictive accuracy of risk tools if a sample is systematically missing the strongest items, but the same logic applies to individual assessments.

### Proration

Another convenient method for handling missing risk assessment data is proration. This technique is performed by averaging an individual’s available items, but it is mathematically equivalent to filling in their missing scores with the mean of their available scores—hence the alternative name *person mean imputation* (Downey & King, 1998; Enders, 2010; Schafer & Graham, 2002). Proration is common in violence risk assessment, with various tools providing proration guidelines, such as the ODARA and DVRAG (Hilton, 2021), Violence Risk Scale-Sexual Offense version (Wong et al., 2003–2020), and Violence Risk Appraisal Guide-Revised (Harris et al., 2015). Similar to mechanical scoring, proration redefines a tool’s composition by the level and pattern of missing data (Schafer & Graham, 2002). It also assumes a given case will score similarly across all scale items (Mazza et al., 2015) and may therefore over- or underestimate risk when this assumption is violated (see Perley-Robertson et al. for a discussion).

### Multiple Imputation

Multiple imputation is an advanced statistical technique widely regarded as one of the best options for addressing missing data (Enders, 2010; Schafer & Graham, 2002). It involves generating multiple datasets with different estimates of the missing values, running the substantive analyses on each dataset, and then aggregating these results. Most imputation algorithms use a two-step iterative regression procedure to fill in the missing values (Enders & Baraldi, 2018). In Step 1, regression equations are constructed from the observed data to predict the missing values, each with an added residual term that maintains the natural variability in scores. In Step 2, these imputed values are used to estimate new regression parameters, which are carried forward to the next two-step iteration. This process is repeated until parameter estimate distributions from Step 2 are stable across iterations (Enders, 2010). Multiple imputation assumes data are at least missing at random (MAR; Rubin, 1987) and yields more accurate parameter estimates and standard errors than traditional techniques when data are not missing completely at random (MCAR; Baraldi & Enders, 2010; de Goeij et al., 2013; Schafer & Graham, 2002; Woods et al., 2023). For more information on this technique, see the online supplementary material.

## Present Study

Researchers and practitioners have employed various methods to address missing data in IPV risk assessment, yet there has been little empirical investigation into the impact of these approaches on predictive accuracy. Our study builds on Perley-Robertson and colleagues’ (2024) initial work on this topic in two important ways. First, we examined how missing data affected the ODARA, a widely used IPV risk assessment tool not previously studied in this context. Second, we addressed a key limitation of their research by applying the same five generated missing data conditions not only to the full sample, but also to two randomly selected subsets comprising one third and two thirds of the sample. This will determine whether introducing measurement error for some versus all cases (and in different amounts) affects relative predictive accuracy.

Based on Perley-Robertson et al.’s findings, we expected mechanical scores to decrease as the missing data rate increased but for prorated and multiply imputed scores to remain stable. We also hypothesized that neither the missing data rate nor the handling method would affect relative predictive accuracy when the missing data conditions were applied to the full sample. We expected similar results for proration and multiple imputation when missing data generation was applied to the sample subsets, as these techniques preserved average absolute risk estimates. However, as mechanical scoring underestimated absolute risk, we expected this method to produce relative predictive accuracy estimates that diverged from the original results when data were deleted for different sample subsets.

## Method

### Sample

The original sample included 300 men charged with an assault or other violent offense against a woman who was their intimate partner. Their case files were subsequently referred to a specialized police service in Canada for a comprehensive threat assessment between 2010 and 2016 (for more information on the sampling context, see Ennis et al., 2015). Cases who were missing a release date or recidivism information were excluded from the study (*n* = 33), resulting in a final sample of 267.

The average age at the index offense was 34.8 (*SD* = 8.9; range = 18 to 65). Most men were identified in the threat assessment records as White (64%, *n* = 168) or Indigenous (27%, *n* = 73), with the remaining identified as other ethnicities (9%, *n* = 23; 1% missing, *n* = 3). On average, the highest grade completed at the time of the index offense was 10.5 (*SD* = 2.2). Only 35% (*n* = 94) of the men completed high school by this point and 52% (*n* = 115) were unemployed. Half of the men (51%, *n* = 136) were married or cohabiting with the victim at the time of the index offense, 28% (*n* = 75) were formerly married or cohabiting, 12% (*n* = 33) were currently dating but living separately, and 8% (*n* = 20) were previously dating (1% missing, *n* = 3). Most of these relationships (62%, *n* = 166) lasted at least 24 months.

The original use of this dataset was approved by the research ethics boards of Pham and colleagues’ (2023) four primary institutions. A formal notice of collaboration was obtained from the law enforcement agency where the study took place.

### Measures

#### Ontario Domestic Assault Risk Assessment

The ODARA is a 13-item actuarial tool originally designed to predict IPV recidivism in men who committed an assault against a woman in a current or former domestic relationship (Hilton et al., 2004). Items are rated on a 2-point scale (0 = *absent*, 1 = *present*) and concern criminal history, index offense details, victim circumstances, and the perpetrator’s and/or victim’s children. Total scores are calculated by summing the items, with proration permitted for a maximum of five ambiguous items (Hilton, 2021); however, in the current study, we calculated prorated scores for any number of omitted items to examine the full effect of missing data. ODARA assessments were completed retrospectively by trained research assistants using threat assessment files (Pham et al., 2023). These included the threat assessment referral form, threat assessment report, and police documents describing the index IPV offense that led to the referral. Referral forms included index offense information and the perpetrator’s IPV offense history. Threat assessment reports, completed by certified threat assessors, covered employment history, childhood history, substance abuse history, relationship history, IPV offense history, and criminal convictions/sentences. These reports also typically included a completed risk assessment tool, such as the ODARA, SARA-V2, SARA-V3, or a combination of the ODARA and one version of the SARA. Police documents included investigator notes, evidence, and arrest details. Other documents that were less consistently available included victim interviews and mental health reports from external agencies. Meta-analytic research shows that the ODARA predicts IPV recidivism with a medium effect size (area under the curve [AUC] = .69; van der Put et al., 2019). More recent research also found the ODARA to predict IPV recidivism among racially diverse samples with small effect sizes (Hegel et al., 2022; Radatz & Hilton, 2022), although further research is needed with Black individuals (Hilton & Radatz, 2024).

#### Spousal Assault Risk Assessment–Version 2

The SARA-V2 (Kropp & Hart, 2000) is a 20-item structured professional judgment tool designed to predict IPV recidivism in people who have committed an IPV offense. Items are rated on a 3-point scale (*no/absent*, *possibly/partially present*, *yes/present*) and concern criminal history, psychosocial adjustment, spousal assault history, and the index IPV offense. These ratings are then used to aid clinical judgment when classifying individuals into one of three summary risk categories (low, moderate, high). SARA-V2 assessments in the current study were completed retrospectively by trained research assistants using the same threat assessment files described above for the ODARA (Pham et al., 2023). Items were coded as “0” if no information indicated they were present or partially present. This approach was based on the assumption that threat assessors documented all relevant information in their reports, which covered many areas assessed by the SARA-V2 and often included a SARA-V2 or SARA-V3 assessment itself. A more conservative approach was taken for the ODARA due to its stricter rules for handling missing items. We discuss the SARA-V2 coding approach in the context of our findings in the Discussion section. In a recent systematic review, mechanically summed SARA-V2 total scores predicted IPV recidivism with a medium effect size (weighted summary AUC = .63; Allard et al., 2024).

#### Recidivism

Recidivism was defined as a new charge or conviction for a violent offense against a current or former intimate partner (conviction data were used for 9% of the sample without charge information). The follow-up period started at charge date for those who were not convicted, conviction date for those with noncustodial sentences, and release date for those with custodial sentences. Charges and convictions were recorded up until July 6, 2020, from local, provincial, and federal sources. Time to recidivism was measured using charge dates when available (91%); otherwise, conviction dates were used (9%). The average length of follow-up was 2.4 years (*SD* = 1.9). During this time, 37% (*n* = 99) of the sample was charged with or convicted of a new IPV offense, and the average time to recidivism was 1.1 years (*SD* = 1.3). For more information on the coding of recidivism data and other procedural details, see Pham et al. (2023).

### Data Analysis Plan

#### Generating Missing Data Conditions

To examine the influence of missing data on predictive accuracy estimates, we used six missing data conditions across three missing data scenarios. Condition 1 was the observed data, which were either complete (SARA-V2) or virtually complete (ODARA). Conditions 2 to 6 were the generated missing data conditions, whereby 1%–50% of MCAR data were inserted into the observed data in 10% increments following Perley-Robertson et al. (2024; 1–10, 11–20, 21–30, 31–40, 41–50). In Scenarios 1 and 2, respectively, one third and two thirds of the sample were randomly selected for missing data generation. In Scenario 3, the entire sample was used for missing data generation (this method was used by Perley-Robertson et al., 2024). This will determine whether introducing measurement error for some versus all cases (and in different amounts) affects relative predictive accuracy. Missing data generation was performed separately by scale using the *prodNA()* function in the missForest package in R (Stekhoven & Bühlmann, 2012).

#### Missing Data Handling Techniques

Mechanical total scores were calculated by summing the available items. Prorated total scores were calculated by averaging the available items and multiplying this by the total number of scale items (Enders, 2010). Multiple imputation was conducted at the item level using chained equations (also called fully conditional specification or sequential regression imputation; Raghunathan et al., 2001; van Buuren & Groothuis-Oudshoorn, 2011). Multiply imputed total scores were then calculated by summing scale items within each imputed dataset.

Chained equations is a common technique for imputing categorical variables that has performed well in numerous simulation studies (Giorgi et al., 2008; Kropko et al., 2014; Moons et al., 2006; Raghunathan et al., 2001; van Buuren et al., 2006). In the current study, chained equations method was conducted in R using the *multivariate imputation by chained equations* (MICE) package (van Buuren & Groothuis-Oudshoorn, 2011). Following recommendations by methodologists, we used a minimum of 20 imputations to meet power requirements (Graham et al., 2007) or matched the number of imputations to the percentage of cases with incomplete data when greater than 20% (see Table S1 of the online supplemental material; Bodner, 2008; White et al., 2011). See the Multiple Imputation section of the online supplemental material for more information on this analytic technique, as well as the diagnostic checks performed.

Recall that multiple imputation assumes the data are MAR. This assumption can be satisfied by incorporating missing data correlates into the imputation phase (Baraldi & Enders, 2010; Collins et al., 2001; Enders, 2010). Methodologists also recommend including correlates of the incomplete variables themselves in the imputation phase to help recover some of the lost information. Together, correlates of missingness and correlates of the incomplete variables are referred to as auxiliary variables. Imputation strategies that make minimal use of auxiliary variables are called *restrictive*, whereas those that make liberal use of auxiliary variables are called *inclusive* (Collins et al., 2001).

Simulation research has demonstrated the benefits of inclusive imputation strategies, including the reduced chance of inadvertently omitting a cause of missingness, reduced bias, and increased power (Collins et al., 2001). However, Perley-Robertson et al. (2024) found that restrictive and inclusive imputation models produced virtually identical relative predictive accuracy estimates. Given these findings and the fact that our data were either complete (SARA-V2) or virtually complete (ODARA), we did not include auxiliary variables in our models. This decision also extended to the exclusion of recidivism as an auxiliary variable. Although methodologists advocate for using the outcome variable to predict missing values, this imputation approach is inappropriate for prognostic assessments (for a discussion, see Perley-Robertson et al., 2024).

#### Preservation of Risk Scores

To examine the preservation of ODARA and SARA-V2 scores across missing data conditions and scenarios, we used descriptive statistics. This included means, standard deviations, and the percentage of change in mean scale scores from Condition 1 (observed data) to Condition 6 (41%–50% generated missing data).

#### Relative Predictive Accuracy

We used Harrell’s concordance index (c-index; Harrell et al., 1982) to examine the relative predictive accuracy of the ODARA and SARA-V2. The c-index is the recommended effect size metric in risk assessment when the follow-up period is variable because it accounts for time at risk to reoffend (Helmus & Babchishin, 2017). In this study, it represents the probability that of two randomly selected individuals (at least one of whom reoffended), the one with the higher risk score reoffended first. The c-index can vary from 0 to 1, with .50 indicating no predictive discrimination (Harrell et al., 1996). Values of .56, .64, and .71 represent small, medium, and large effect sizes, respectively (Helmus & Babchishin, 2017). The c-index was obtained through Cox regression in R. As described by Pham and colleagues (2023), the proportionality of hazards assumption was met for both scales.

Differences in predictive accuracy within and across missing data conditions/scenarios were examined using confidence intervals and magnitude cut-offs of .56, .64, and .71. Namely, a missing data handling technique was considered meaningfully better than another if (1) it produced categorically better predictive accuracy, and (2) confidence intervals did not overlap (*p* < .01; Cumming & Finch, 2005). Estimates from Condition 1 were used as the baseline for comparisons.

## Results

### Scale Descriptives

#### Ontario Domestic Assault Risk Assessment

Scale descriptives for the ODARA are displayed in Table S2 of the online supplemental material. The overall missing data rate was 1.5%, and the percentage of missing data by item ranged from 0.0% to 8.2%. Across the entire sample, one third of items were scored as 0 (absent), while two thirds were scored as 1 (present). This shows that the sample generally exhibited high levels of IPV recidivism risk. However, the equal item means assumption of proration—that a given respondent will score similarly across items—may still be violated, as two items had even score distributions (*threatened to harm/kill anyone at index assault* and *victim has biological child with previous partner*) and two were more often absent than present (*confinement of victim at index* assault and *ever assaulted victim while she was pregnant*). Table 1 presents the overall missing data rate for the different missing data conditions within each scenario, while Tables S3 to S5 of the online supplemental material show the percentage of missing data by item.

**Table 1.** Overall Missing Data Rates for the ODARA and SARA-V2 Across Missing Data Conditions and Scenarios.

Portion of sample with generated missing data	Condition 1 (observed data)	Condition 2 (1%–10%)	Condition 3 (11%–20%)	Condition 4 (21%–30%)	Condition 5 (31%–40%)	Condition 6 (41%–50%)
ODARA						
Scenario 1: one third	1.5	4.1	6.6	10.4	14.1	16.7
Scenario 2: two thirds	1.5	6.6	11.6	19.1	26.6	31.7
Scenario 3: three thirds	1.5	9.0	16.8	27.8	39.4	47.0
SARA-V2						
Scenario 1: one third	0.0	2.5	5.8	9.1	12.5	15.8
Scenario 2: two thirds	0.0	4.9	11.6	18.3	24.9	31.6
Scenario 3: three thirds	0.0	7.3	17.3	27.3	37.3	47.3

*Note. N* = 267. Missing data rates = $\frac{\mathrm{Total}\;\mathrm{no}.\;\mathrm{missing}\;\mathrm{items}\;\mathrm{across}\;267\;\mathrm{cases}}{\mathrm{Total}\;\mathrm{no}.\;\mathrm{possible}\;\mathrm{items}\;\mathrm{across}\;267\;\mathrm{cases}\;(267\;*\;\mathrm{no}.\;\mathrm{scale}\;\mathrm{items})}$ . Missing Data Scenarios 1 and 2: one third (*n* = 89) and two thirds (*n* = 178) of the sample, respectively, were randomly selected for missing data generation. Missing Data Scenario 3: the entire sample was used for missing data generation. Conditions 2 to 6 represent the percentage of items that were randomly deleted for each case in the scenario sample. ODARA = Ontario Domestic Assault Risk Assessment; SARA-V2 = Spousal Assault Risk Assessment–Version 2.

#### Spousal Assault Risk Assessment–Version 2

Scale descriptives for the SARA-V2 are displayed in Table S6 of the online supplemental material. The overall distribution of item scores was bimodal: 32% of items were scored as 0 (no/absent), 21% as 1 (possibly/partially present), and 47% as 2 (yes/present). This shows that the sample generally exhibited high levels of IPV recidivism risk. Still, proration’s equal item means assumption may be violated, as six means were categorized as no/absent (0.15 to 0.95) and 14 as possibly/partially present (1.06 to 1.89). Table 1 presents the overall missing data rate for the different missing data conditions within each scenario, while Tables S7 to S9 of the online supplemental material show the percentage of missing data by item.

### Preservation of Risk Scores

Depending on the missing data scenario, average mechanical ODARA scores decreased by 15% to 47% from Conditions 1 to 6 (see Table 2). Conversely, average prorated and multiply imputed scores were either unchanged or showed minimal variations (±1% for proration and -1%–4% for multiple imputation). Table 3 shows a similar pattern for the SARA-V2: mechanical scores decreased by 16% to 48% from Conditions 1 to 6, prorated scores were either unchanged or decreased by less than 1%, and multiply imputed scores showed minimal decreases (1%–4%). Hence, proration and multiple imputation preserved total scores as missing data increased, whereas mechanical scoring led to marked reductions in these absolute risk estimates.

**Table 2.** Preservation of ODARA Scores Across Missing Data Conditions and Scenarios by Missing Data Handling Technique.

	*M* (*SD*) scale total score						% change in *M* score from Conditions 1 to 6
Missing data handling technique	Condition 1 (observed data)	Condition 2 (1%–10%)	Condition 3 (11%–20%)	Condition 4 (21%–30%)	Condition 5 (31%–40%)	Condition 6 (41%–50%)	
Missing Data Scenario 1: one third of the sample (*n* = 89) was randomly selected for missing data generation							
Mechanical total	8.6 (2.1)	8.3 (2.1)	8.1 (2.1)	7.8 (2.3)	7.5 (2.4)	7.3 (2.6)	–15.1%
Proration	8.7 (2.2)	8.7 (2.2)	8.7 (2.2)	8.7 (2.2)	8.8 (2.3)	8.8 (2.3)	+1.2%
Multiple imputation	8.7 (2.1)	8.7 (2.1)	8.7 (2.1)	8.7 (2.1)	8.7 (2.1)	8.7 (2.1)	0.0%
Missing Data Scenario 2: two thirds of the sample (*n* = 178) was randomly selected for missing data generation							
Mechanical total	8.6 (2.1)	8.1 (2.0)	7.7 (2.1)	7.0 (2.1)	6.3 (2.3)	6.0 (2.4)	–30.2%
Proration	8.7 (2.2)	8.9 (2.2)	8.7 (2.3)	8.7 (2.3)	8.7 (2.3)	8.7 (2.6)	0.0%
Multiple imputation	8.7 (2.1)	8.7 (2.2)	8.6 (2.2)	8.5 (2.1)	8.5 (2.1)	8.6 (2.2)	–1.2%
Missing Data Scenario 3: the entire sample (*N* = 267) was used for missing data generation							
Mechanical total	8.6 (2.1)	7.9 (2.0)	7.3 (1.9)	6.3 (1.7)	5.3 (1.5)	4.6 (1.4)	–46.5%
Proration	8.7 (2.2)	8.9 (2.3)	8.7 (2.3)	8.8 (2.3)	8.8 (2.4)	8.6 (2.5)	–1.2%
Multiple imputation	8.7 (2.1)	8.7 (2.1)	8.6 (2.1)	8.6 (2.1)	8.6 (2.1)	8.4 (2.0)	–3.5%

*Note.* All analyses were run on the full sample. Conditions 2 to 6 represent the percentage of items that were randomly deleted for each case in the scenario sample. ODARA = Ontario Domestic Assault Risk Assessment.

**Table 3.** Preservation of SARA-V2 Scores Across Missing Data Conditions and Scenarios by Missing Data Handling Technique.

	*M* (*SD*) scale total score						% change in *M* score from Conditions 1 to 6
Missing data handling technique	Condition 1 (observed data)	Condition 2 (1%–10%)	Condition 3 (11%–20%)	Condition 4 (21%–30%)	Condition 5 (31%–40%)	Condition 6 (41%–50%)	
Missing Data Scenario 1: one third of the sample (*n* = 89) was randomly selected for missing data generation							
Mechanical total	23.1 (5.7)	22.4 (5.7)	21.6 (5.8)	21.0 (6.0)	20.2 (6.5)	19.4 (7.2)	–16.0%
Proration	–	22.9 (5.8)	23.0 (5.9)	23.1 (5.7)	23.1 (5.9)	23.1 (6.2)	0.0%
Multiple imputation	–	23.0 (5.7)	23.0 (5.7)	23.0 (5.6)	22.9 (5.6)	22.9 (5.8)	–0.9%
Missing Data Scenario 2: two thirds of the sample (*n* = 178) was randomly selected for missing data generation							
Mechanical total	23.1 (5.7)	21.9 (5.5)	20.3 (5.5)	18.8 (5.6)	17.4 (5.9)	15.8 (6.4)	–31.6%
Proration	–	23.0 (5.7)	23.1 (5.9)	23.1 (6.1)	23.3 (6.3)	23.1 (6.2)	0.0%
Multiple imputation	–	23.0 (5.7)	22.9 (5.7)	22.9 (5.8)	22.9 (5.8)	22.7 (5.4)	–1.7%
Missing Data Scenario 3: the entire sample (*N* = 267) was used for missing data generation							
Mechanical total	23.1 (5.7)	21.3 (5.3)	19.0 (5.0)	19.0 (5.0)	14.4 (3.9)	12.1 (3.6)	–47.6%
Proration	–	23.0 (5.7)	23.1 (6.1)	23.1 (6.1)	23.0 (6.2)	23.0 (7.0)	–0.4%
Multiple imputation	–	22.9 (5.6)	22.8 (5.8)	22.8 (5.8)	22.5 (5.5)	22.3 (5.8)	–3.5%

*Note.* All analyses were run on the full sample. Condition 1 data were complete, so only mechanical scoring was used to calculate the total score. This score (23.1) served as the starting point for all comparisons in the rightmost column (% change in *M* score from Conditions 1 to 6). Conditions 2 to 6 represent the percentage of items that were randomly deleted for each case in the scenario sample. SARA-V2 = Spousal Assault Risk Assessment–Version 2.

### Relative Predictive Accuracy

For the ODARA, mechanical scoring in Condition 1 (observed data) produced a nonsignificant c-index of .55, while proration and multiple imputation both yielded significant c-indexes of .56 (see Table 4). For the SARA-V2, mechanical scoring in Condition 1 produced a nonsignificant c-index of .56 (see Table 5). Deviations from these original values were small for all three missing data handling techniques, regardless of whether the missing data conditions (1%–10%, 11%–20%, 21%–30%, 31%–40%, 41%–50%) were applied to one third, two thirds, or three thirds of the dataset. When expressed as percentages, multiple imputation produced c-indexes that deviated from the original results by 0–2 percentage points for the ODARA and 0–1 percentage point for the SARA-V2. For both scales, proration and mechanical scoring produced c-indexes that deviated from the original results by 0–3 percentage points. There were slight variations in statistical significance with no clear pattern, and confidence intervals overlapped across all missing data conditions and scenarios. This indicates that mechanical scoring, proration, and multiple imputation discriminated recidivists from nonrecidivists with comparable accuracy (*p* > .01).

**Table 4:** Relative Predictive Accuracy of the ODARA Across Missing Data Conditions and Scenarios by Missing Data Handling Technique.

	Harrell’s c-index [95% CI]					
Missing data handling technique	Condition 1 (observed data)	Condition 2 (1%–10%)	Condition 3 (11%–20%)	Condition 4 (21%–30%)	Condition 5 (31%–40%)	Condition 6 (41%–50%)
Missing Data Scenario 1: one third of the sample (*n* = 89) was randomly selected for missing data generation						
Mechanical total	.55 [.496, .611]	.55 [.497, .610]	.55 [.489, .603]	.55 [.493, .608]	.53 [.477, .592]	.54 [.484, .597]
Proration	**.56 [.506, .620]**	**.57 [.509, .623]**	.55 [.497, .612]	**.56 [.507, .622]**	.56 [.499, .614]	**.58 [.520, .637]**
Multiple imputation	**.56 [.502, .617]**	**.56 [.505, .622]**	.56 [.497, .617]	**.56 [.502, .623]**	.56 [.498, .620]	**.57 [.503, .630]**
Missing Data Scenario 2: two thirds of the sample (*n* = 178) was randomly selected for missing data generation						
Mechanical total	.55 [.496, .611]	.56 [.498, .614]	.55 [.495, .611]	**.58 [.525, .641]**	**.56 [.505, .621]**	.55 [.495, .613]
Proration	**.56 [.506, .620]**	.56 [.500, .615]	.55 [.495, .610]	**.58 [.521, .636]**	**.57 [.512, .628]**	.55 [.496, .612]
Multiple imputation	**.56 [.502, .617]**	.56 [.500, .617]	.55 [.490, .607]	**.58 [.517, .638]**	**.56 [.501, .627]**	.56 [.497, .625]
Missing Data Scenario 3: the entire sample (*N* = 267) was used for missing data generation						
Mechanical total	.55 [.496, .611]	**.56 [.504, .618]**	.54 [.484, .600]	.55 [.499, .609]	**.56 [.502, .617]**	.52 [.460, .574]
Proration	**.56 [.506, .620]**	**.57 [.511, .626]**	.55 [.495, .611]	**.56 [.509, .620]**	**.56 [.506, .623]**	.53 [.474, .588]
Multiple imputation	**.56 [.502, .617]**	**.57 [.507, .624]**	.55 [.489, .613]	.56 [.498, .622]	.56 [.490, .623]	.54 [.467, .605]

*Note.* All analyses were run on the full sample. Statistically significant effect sizes are bolded. Conditions 2 to 6 represent the percentage of items that were randomly deleted for each case in the scenario sample. CI = confidence interval; ODARA = Ontario Domestic Assault Risk Assessment.

**Table 5.** Relative Predictive Accuracy of the SARA-V2 Across Missing Data Conditions and Scenarios by Missing Data Handling Technique.

	Harrell’s c-index [95% CI]					
Missing data handling technique	Condition 1 (observed data)	Condition 2 (1%–10%)	Condition 3 (11%–20%)	Condition 4 (21%–30%)	Condition 5 (31%–40%)	Condition 6 (41%–50%)
Missing Data Scenario 1: one third of the sample (*n* = 89) was randomly selected for missing data generation						
Mechanical total	.56 [.499, .621]	**.56 [.503, .624]**	**.57 [.508, .627]**	.56 [.497, .614]	.54 [.484, .610]	.53 [.471, .591]
Proration	–	.56 [.496, .619]	**.56 [.502, .625]**	**.56 [.501, .622]**	**.56 [.501, .621]**	.55 [.486, .607]
Multiple imputation	–	.56 [.499, 622]	.56 [.497, 621]	.56 [.496, 620]	**.56 [.501, 626]**	.55 [.489, .617]
Missing Data Scenario 2: two thirds of the sample (*n* = 178) was randomly selected for missing data generation						
Mechanical total	.56 [.499, .621]	**.57 [.513, .634]**	**.58 [.522, .645]**	**.56 [.501, .621]**	.55 [.490, .612]	.55 [.492, .610]
Proration	–	**.57 [.509, .631]**	**.57 [.512, .636]**	.55 [.492, .612]	.55 [.484, .608]	.56 [.499, .619]
Multiple imputation	–	**.57 [.509, .631]**	.56 [.499, .624]	.56 [.497, .621]	.55 [.488, .618]	.56 [.497, .629]
Missing Data Scenario 3: the entire sample (*N* = 267) was used for missing data generation						
Mechanical total	.56 [.499, .621]	**.56 [.503, .625]**	**.56 [.501, .619]**	.56 [.498, .618]	.53 [.474, .594]	.55 [.483, .607]
Proration	–	.56 [.498, .621]	.56 [.498, .616]	.55 [.495, .614]	.53 [.474, .594]	.54 [.481, .605]
Multiple imputation	–	.56 [.493, .617]	**.56 [.502, .623]**	.55 [.490, .616]	.55 [.484, .616]	.55 [.484, .620]

*Note.* All analyses were run on the full sample. Statistically significant effect sizes are bolded. Conditions 2 to 6 represent the percentage of items that were randomly deleted for each case in the scenario sample. CI = confidence interval; SARA-V2 = Spousal Assault Risk Assessment–Version 2.

## Discussion

Missing data are pervasive in IPV risk assessment, but there is limited evidence about their impact on predictive accuracy. The most common techniques used to address missing risk assessment data are mechanical scoring (summing available items) and proration (averaging available items). Although both approaches can be used in practice and research, multiple imputation is considered preferable in research contexts due to its statistical advantages demonstrated in the broader missing data literature (e.g., Hildebrand et al., 2013; Kroner & Yessine, 2013; Whiting et al., 2023).

However, the first study comparing these three techniques in correctional risk assessment did not show multiple imputation to be superior—at least not when compared to proration (Perley-Robertson et al., 2024). A notable limitation of this study, though, was the uniform deletion of items across cases, which may have preserved relative predictive accuracy estimates. Our research addressed this limitation by comparing the performance of mechanical scoring, proration, and multiple imputation when data were deleted across the entire sample and, importantly, in different sample subsets. We also re-examined the SARA-V2 and extended our investigation to the ODARA, advancing the research on two of the most commonly used IPV risk assessment tools in Canada (Goossens et al., 2024).

As expected, average prorated and multiply imputed scores for both tools showed only small fluctuations from Conditions 1 to 6 (0%–4%), whereas average mechanical scores decreased by 15% to 48%. Hence, simply summing the available items substantially underestimated absolute risk estimates. In examining the relative predictive accuracy of the ODARA and SARA-V2, our hypotheses were partially supported. Neither the missing data rate nor the handling method affected relative predictive accuracy when missing data conditions were applied to the full sample, confirming our predictions. The same results were found for proration and multiple imputation when missing data conditions were applied to sample subsets, also as we expected (note that for all analyses, the potential violation of proration’s equal item means assumption did not introduce bias into the results). The surprising finding came from mechanical scoring, which did not impact predictive accuracy estimates when data were deleted for different sample subsets.

### Strengths, Limitations, and Future Research Directions

Previous research examining the effect of missing data on IPV risk tools was restricted to men with dual IPV and sexual offending histories, which included intimate partner sexual violence for some (Perley-Robertson et al., 2024). Men who commit such offenses have distinct career paths that are more specialized in sexual offending than IPV (Chopin et al., 2025) and may pose a higher risk of IPV recidivism than men with no sexual offense history (Sparks et al., 2020). A primary strength of our study is that we extend Perley-Robertson et al.’s results to a sample that more closely approximates the typical IPV offending population: individuals with a history of IPV, regardless of sexual violence. Nevertheless, our sample comprised relatively high-risk men referred for specialized threat assessment (Hilton et al., 2021), potentially limiting the generalizability of results to routine police caseloads.

It is also important to note that our findings attest only to the trivial impact of random missingness (as opposed to systematic missingness) on the relative predictive accuracy of IPV risk tools. This limitation is significant because real-world data are rarely MCAR (Raghunathan, 2004). In Canadian provincial corrections, for example, psychological assessments might only be available when there is enough cause for concern to request them (Perley-Robertson et al., 2024). Research is, therefore, needed to examine the effect of missing data on the relative predictive accuracy of IPV risk tools under conditions that reproduce these more realistic scenarios.

Another important limitation is that we generated missing data with equal likelihood across all items, but some items may contribute more to the prediction of IPV recidivism than others. For example, a large meta-analysis comprising 105 unique samples found that the perpetrator’s criminal history is a stronger predictor of domestic violence recidivism than information about the index assault (Perley-Robertson et al., 2025). Given this variability in predictive power, future research should examine the relative impact of deleting the strongest versus weakest items from IPV risk tools. This would test whether some items can tolerate more missing data than others, potentially informing assessors about when proration is appropriate versus when additional information should be sought. Unfortunately, we could not test whether deleting strong versus weak items would affect predictive accuracy because effect sizes for total scores were already low (i.e., item-level predictive validity would likely also be low because total scores usually produce larger effect sizes than individual items; e.g., see Giguère et al., 2023). Other forms of systematic missingness also warrant investigation in this understudied area. For example, items could be deleted based on perpetrator risk level or typology, or based on scale psychometric properties, such as inter-rater reliability, item difficulty, or factor structure.

We used the SARA-V2 instead of the newer SARA-V3, which is intended to help evaluators exercise their professional judgment rather than score items and interpret a risk scale score (Kropp & Hart, 2015). The SARA-V3 has several additional items, such as major mental disorder and a six-item section on victim vulnerability factors, which is intended to be based on the psychosocial adjustment of the primary victim (Kropp & Hart, 2015). It is possible that the SARA-V3 may be more extensively compromised by systematically missing data than the SARA-V2 because of these additional items. As well as mental health being challenging to score in the policing context (Hilton et al., 2021; Jung & Buro, 2017), victim vulnerability information may be missing if the victim is unavailable or unamenable to participating in the risk assessment. Gray and colleagues (2025) reported that 40% of their correctional sample was missing the ODARA item victim concern and recommended that future research find behavioral indicators to serve as proxy measures for this item. However, examining the impact of specific missing items on predictive accuracy may be a beneficial first step.

Furthermore, we tested the predictive accuracy of the ODARA and SARA-V2 using a rank ordering statistic. The observation that effect sizes remained stable with mechanical scoring despite a substantial reduction in absolute risk illustrates that discrimination (distinguishing recidivists from nonrecidivists through relative risk estimates) and calibration (correctly predicting recidivism rates through absolute risk estimates) are distinct characteristics of risk assessment tools (Hanson, 2017). Research is therefore needed to examine the effect of missing data and corresponding handling techniques on calibration statistics. Findings would have implications for the interpretation of risk estimates produced by actuarial IPV tools, such as the ODARA and DVRAG.

Research is also needed using complete assessments to examine the full effect of missing data on the predictive accuracy of correctional risk tools. In the only previous study on this topic, the STABLE-2007 and SARA-V2 had overall missing data rates of 1% and 16% prior to data cleaning, respectively (Perley-Robertson et al., 2024). In the current study, the ODARA had an overall missing data rate of 1.5%, while the SARA-V2 appeared complete because Pham and colleagues (2023) coded items as absent (rather than missing) when no information indicated their presence. This approach was based on the assumption that threat assessors documented all relevant information in their reports, which covered many areas assessed by the SARA-V2 and often included a SARA-V2 or SARA-V3 assessment itself.

Although some SARA-V2 items may have been genuinely missing rather than absent, it was not feasible for us to make this distinction. Our SARA-V2 results, therefore, contain an unknown amount of error. However, this error is likely minimal given the low missing data rate for the ODARA and is likely restricted mainly to items that would come from mental health reports (*recent suicidal or homicidal ideation/intent*, *recent psychotic and/or manic symptoms*, *personality disorder*), which were not consistently available in threat assessment files. Nonetheless, the similar pattern of findings between the ODARA and SARA-V2 suggests this error did not affect the reliability of our results.

A further limitation concerns the predictive performance of the ODARA and SARA-V2. Effect sizes for both scales were either small or fell below this threshold. Although Pham and colleagues (2023) reported slightly higher predictive accuracy estimates with this sample, they used AUCs, which do not control for time at risk. Nonetheless, both metrics suggest relatively poor performance compared to other studies (Allard et al., 2024; van der Put et al., 2019). This reduced discriminative ability likely stems from our sample’s homogeneity, comprising high-risk men referred for specialized threat assessments. As the ODARA and SARA-V2 were developed and/or validated using more representative samples (Hilton, 2021; Kropp & Hart, 2000), they may not be as useful for discriminating high-risk recidivists from nonrecidivists. Future research with samples at relatively high risk of recidivism should consider using alternative tools designed for this population (e.g., DVRAG; Hilton, 2021).

### Implications for IPV Risk Assessment

Our study adds to growing evidence that researchers need not delete cases with missing data when examining the predictive accuracy of IPV risk tools. This finding has important methodological implications, as retaining incomplete cases may yield more representative samples, more powerful analyses, and more reproducible results. Moreover, our findings demonstrate that the ODARA and SARA-V2 are robust to large amounts of random missingness. Assessors can therefore be confident that occasional gaps in information will not compromise the relative predictive accuracy of these scales.

When evaluating individual cases for IPV risk, proration is a more defensible approach than mechanical scoring. This is because proration aligns missing item scores with observed risk levels, whereas mechanical scoring assumes missing items are absent. Unsurprisingly, making such an assumption can lead to the underestimation of absolute risk estimates, which directly inform IPV risk management strategies (Hilton, 2021).

Although our results are promising, we recommend that assessors adhere to official scoring guidelines where they exist. For example, we prorated up to eight ODARA items for research purposes, but only five can be prorated in practice (Hilton, 2021). In research contexts, our study supports prorating up to eight items, though sensitivity analyses following the official scoring guidelines would provide a useful safeguard until more research is conducted. The SARA-V2 manual does not recommend the use of total scores because it is a structured professional judgment tool (Kropp, Hart, Webster, & Eaves, 2008), but scores are reported in various applied settings (Ahmed, 2020; Schafers et al., 2021) and are commonly used for research purposes (Allard et al., 2024). As the user manual does not specify how to handle missing information when calculating SARA-V2 total scores, proration in assessment reports and either proration or multiple imputation in research would be defensible. For tools without missing data guidelines, our findings indicate that proration is a defensible applied approach, whereas multiple imputation offers a more theoretically sound approach for research samples.

## Conclusion

This study provides compelling evidence that the ODARA and SARA-V2 are robust to randomly missing data. Proration and multiple imputation performed optimally, preserving both absolute risk scores and relative predictive accuracy. Mechanical scoring also preserved relative predictive accuracy, but it substantially underestimated absolute risk. Hence, researchers can retain incomplete cases in predictive validity studies and calculate either prorated or multiply imputed scores, potentially yielding more representative samples and powerful analyses. However, in practice, we recommend that assessors follow official scoring guidelines for IPV risk assessment tools. We also encourage scale developers to examine the benefits of adding proration guidelines to their tools if not already included. Future research should examine the impact of systematic missingness on the relative and absolute predictive accuracy of IPV risk tools, and whether some items can better tolerate missing data.

## Supplemental Material

sj-docx-1-asm-10.1177_10731911251386519 – Supplemental material for The Effect of Missing Item Data on the Relative Predictive Accuracy of Intimate Partner Violence Risk Assessment Tools Supplemental material, sj-docx-1-asm-10.1177_10731911251386519 for The Effect of Missing Item Data on the Relative Predictive Accuracy of Intimate Partner Violence Risk Assessment Tools by Bronwen Perley-Robertson, Anna T. Pham and N. Zoe Hilton in Assessment

## Data Availability

Data for this study are not publicly available.
