# Supplementary material for: The Effect of Missing Item Data on the Relative Predictive Accuracy of Intimate Partner Violence Risk Assessment Tools
Source: Assessment. 2025 Nov 12;33(8):1320–33. doi: 10.1177/10731911251386519 (PMC13675582; doi:10.1177/10731911251386519)
Supplement: Supplementary file 1 [file sj-docx-1-asm-10.1177_10731911251386519.docx]

**The Effect of Missing Item Data on the Relative Predictive Accuracy of Intimate Partner**

# Violence Risk Assessment Tools

Online Supplemental Material

Bronwen Perley-Robertson, Anna T. Pham, and N. Zoe Hilton

# Table of Contents

Multiple Imputation ........................................................................................................................ 1

Model Details ........................................................................................................................... 2

Table S1: Number of Multiply Imputed Datasets for Each Model .................................. 2

Model Diagnostics ................................................................................................................... 3

References ....................................................................................................................................... 4

Supplementary ODARA Tables ..................................................................................................... 5

Table S2: ODARA Scale Descriptives for the Observed Data (Distribution of Item

Scores) ..................................................................................................................... 5

Table S3: ODARA Missing Data Scenario 1 (Data Deleted for 1/3 of the Sample):

Number and Percentage of Missing Items by Missing Data Condition .................. 6

Table S4: ODARA Missing Data Scenario 2 (Data Deleted for 2/3 of the Sample):

Number and Percentage of Missing Items by Missing Data Condition .................. 7

Table S5: ODARA Missing Data Scenario 3 (Data Deleted for 3/3 of the Sample):

Number and Percentage of Missing Items by Missing Data Condition .................. 8

Supplementary SARA-V2 Tables ................................................................................................... 9

Table S6: SARA-V2 Scale Descriptives for the Observed Data (Distribution of

Item Scores and Item Means) .................................................................................. 9

Table S7: SARA-V2 Missing Data Scenario 1 (Data Deleted for 1/3 of the Sample):

Number and Percentage of Missing Items by Missing Data Condition ................ 10

Table S8: SARA-V2 Missing Data Scenario 2 (Data Deleted for 2/3 of the Sample):

Number and Percentage of Missing Items by Missing Data Condition ................ 11

Table S9: SARA-V2 Missing Data Scenario 3 (Data Deleted for 3/3 of the Sample):

Number and Percentage of Missing Items by Missing Data Condition ................ 12

# Multiple Imputation

Multiple imputation was conducted at the item level using chained equations (i.e., fully conditional specification or sequential regression imputation; Raghunathan et al., 2001; van Buuren et al., 2006; van Buuren & Groothuis-Oudshoorn, 2011). As summarized by PerleyRobertson et al. (2024), this technique fills in missing data one variable at a time using an iterative process that matches the imputation model to each variable’s distributional form. For instance, linear regression is used to impute continuous variables, logistic regression is used to impute binary variables, and proportional odds logistic regression is used to impute ordinal variables. Each regression model defines a distribution of plausible replacement values for the incomplete variable based on the predictors and a residual term. Replacement values are then drawn at random from this distribution. This imputation process is performed sequentially, starting with the most complete variable, and each sequence of imputations informs the next (i.e., after an incomplete variable is imputed, it is used as a predictor for every other incomplete variable in the chain).

Once the entire dataset is filled in, a Bayesian procedure is used to sample a new set of regression parameters, which are used to generate the next set of imputations. Of note, each iteration uses the imputations from the previous cycle to predict missing values. This process continues until the distributions of the parameter estimates are stable across iterations (i.e., convergence), with the final iteration providing the data for analysis. Imputing data this way provides a good approximation of what they would look like had they been complete (Baraldi & Enders, 2010; Enders, 2010; Newman, 2014; Woods, 2023). Aggregating results across multiply imputed datasets also accounts for uncertainty in missing values (Baraldi & Enders, 2010).

In the current study, chained equations was conducted in R using the *multivariate imputation by chained equations* (MICE) package (van Buuren & Groothuis-Oudshoorn, 2011). MICE employs parallel data augmentation, wherein *m* imputation chains that run for *t* iterations generate one imputed dataset each. This contrasts sequential data augmentation, wherein one long imputation chain saves *m* imputed datasets at every *t*th iteration (Enders, 2010).

**Model Details**

Following recommendations by methodologists, we used a minimum of 20 imputations to meet power requirements (Graham et al., 2007), or matched the number of imputations to the percentage of cases with incomplete data when greater than 20% (see Table S1; Bodner, 2008; White et al., 2011). Additionally, we used 100 iterations for each imputation model.

**Table S1** *Number of Multiply Imputed Datasets for Each Model*

| Missing data scenario/condition | ODARA | SARA-V2 |
| --- | --- | --- |
| Condition 1 (observed data) | 20 (17% of cases incomplete) | NA (no missing data) |
| Scenario 1: Conditions 2–6 | 45 | 33 |
| Scenario 2: Conditions 2–6 | 73 | 67 |
| Scenario 3: Conditions 2–6 | 100 | 100 |

*Note.* Missing Data Scenarios 1 and 2: one-third (*n* = 89) and two-thirds (*n* = 178) of the sample, respectively, were randomly selected for missing data generation. Missing Data Scenario 3: the entire sample was used for missing data generation. Conditions 2 to 6 represent the generated missing data conditions. ODARA = Ontario Domestic Assault Risk Assessment; SARA-V2 = Spousal Assault Risk Assessment - Version 2.

**Model Diagnostics**

Model convergence was assessed using trace and potential scale reduction (PSR) factor plots. As summarized by Perley-Robertson et al. (2024), trace plots in MICE display the mean and standard deviation for each imputed variable in the *m* chains plotted against the iteration number (van Buuren & Groothuis-Oudshoorn, 2011). Convergence is reached when there are no systematic changes in the parameters across iterations and the chains are intermingled. The PSR factor is a numeric diagnostic of the stability of parameter estimates across iterations (Gelman & Rubin, 1992). It essentially measures how much the imputations will improve with more iterations. Convergence is reached when PSR factor values are close to 1 (1.1 is the recommended threshold). Distributional discrepancy—the difference between observed and imputed data—was examined using density plots, which display the kernel density estimates (i.e., distribution) of the observed and imputed data (van Buuren, 2018). Distributions that align give a good indication that imputed data are plausible (van Buuren provides an example of a density plot for a categorical variable; see Section 6.6.2).

Trace plots for both the ODARA and SARA-V2 showed no systematic changes in means or standard deviations across iterations and chains were intermingled. For the ODARA, most PSR factor values were below 1.1 immediately or within a small number of iterations (5–15), whereas others dropped below this threshold by the 40^th^ iteration. For the SARA-V2, most PSR factor values were below 1.1 immediately or within a small number of iterations (5–10), whereas others dropped below this threshold by the 25^th^ iteration. Density plots for both scales showed good alignment between observed and imputed values, indicating no distributional discrepancies.

# References

* Additional references not in the manuscript.

Gelman, A., & Rubin, D. B. (1992). Inference from iterative simulation using multiple sequences. *Statistical Science*, *7*(4), 457–472. <https://doi.org/10.1214/ss/1177011136>

Newman, D. A. (2014). Missing data: Five practical guidelines. *Organizational Research*

*Methods*, *17*(4), 372–411. https://doi.org/10.1177/1094428114548590 van Buuren, S. (2018). *Flexible imputation of missing data* (2nd ed.). Chapman and Hall/CRC.

<https://stefvanbuuren.name/fimd/>

**2**

*Scale Descriptives for the Observed Data (Distribution of Item Scores)*

|  |  | Item score |  |
| --- | --- | --- | --- |
| ODARA item | 0: *n* (%) | 1: *n* (%) | Missing: *n* (%) |
| Pre-index domestic assault in police/criminal record | 20 (7.5) | 247 (92.5) | 0 (0.0) |
| Pre-index nondomestic assault in police /criminal record | 62 (23.2) | 203 (76.0) | 2 (0.7) |
| Pre-index custodial sentence of 30 days or more | 110 (41.2) | 156 (58.4) | 1 (0.4) |
| Failure on pre-index conditional release | 40 (15.0) | 227 (85.0) | 0 (0.0) |
| Threat to physically harm or kill at index assault | 132 (49.4) | 132 (49.4) | 3 (1.1) |
| Confinement of the partner at index assault | 189 (70.8) | 76 (28.5) | 2 (0.7) |
| Victim concern about future assaults | 67 (25.1) | 181 (67.8) | 19 (7.1) |
| More than one child altogether | 78 (29.2) | 189 (70.8) | 0 (0.0) |
| Victim’s biological child from a previous partner | 126 (47.2) | 140 (52.4) | 1 (0.4) |
| Pre-index violence against a nondomestic victim | 47 (17.6) | 219 (82.0) | 1 (0.4) |
| Two or more indicators of substance abuse | 45 (16.9) | 221 (82.8) | 1 (0.4) |
| Assault on index victim when pregnant | 163 (61.0) | 82 (30.7) | 22 (8.2) |
| Barrier to victim support | 52 (19.5) | 214 (80.1) | 1 (0.4) |
| Overall distribution of item scores | 1,131 (32.6) | 2,287 (65.9) | 53 (1.5) |

*Note.* *N* = 267. Item scores: 0 = *absent*; 1 = *present*; ODARA = Ontario Domestic Assault Risk Assessment.	**3**

*Missing Data Scenario 1 (Data Deleted for 1/3 of the Sample):*

| ODARA item | Condition 1 (observed) | Condition 2 (1%–10%) | Condition 3  (11%–20%) | Condition 4  (21%–30%) | Condition 5  (31%–40%) | Condition 6  (41%–50%) |
| --- | --- | --- | --- | --- | --- | --- |
| Pre-index domestic assault in police/criminal record | 0 (0.0) | 10 (3.7) | 14 (5.2) | 27 (10.1) | 31 (11.6) | 36 (13.5) |
| Pre-index nondomestic assault in police /criminal record | 2 (0.7) | 7 (2.6) | 16 (6.0) | 24 (9.0) | 32 (12.0) | 40 (15.0) |
| Pre-index custodial sentence of 30 days or more | 1 (0.4) | 6 (2.2) | 13 (4.9) | 22 (8.2) | 28 (10.5) | 35 (13.1) |
| Failure on pre-index conditional release | 0 (0.0) | 12 (4.5) | 17 (6.4) | 24 (9.0) | 33 (12.4) | 40 (15.0) |
| Threat to physically harm or kill at index assault | 3 (1.1) | 11 (4.1) | 18 (6.7) | 31 (11.6) | 48 (18.0) | 53 (19.9) |
| Confinement of the partner at index assault | 2 (0.7) | 9 (3.4) | 11 (4.1) | 19 (7.1) | 36 (13.5) | 45 (16.9) |
| Victim concern about future assaults | 19 (7.1) | 24 (9.0) | 31 (11.6) | 45 (16.9) | 57 (21.3) | 64 (24.0) |
| More than one child altogether | 0 (0.0) | 8 (3.0) | 15 (5.6) | 27 (10.1) | 41 (15.4) | 42 15.7) |
| Victim’s biological child from a previous partner | 1 (0.4) | 6 (2.2) | 11 (4.1) | 25 (9.4) | 37 (13.9) | 48 (18.0) |
| Pre-index violence against a nondomestic victim | 1 (0.4) | 4 (1.5) | 12 (4.5) | 21 (7.9) | 28 (10.5) | 38 (14.2) |
| Two or more indicators of substance abuse | 1 (0.4) | 9 (3.4) | 18 (6.7) | 24 (9.0) | 37 (13.9) | 44 (16.5) |
| Assault on index victim when pregnant | 22 (8.2) | 31 (11.6) | 37 (13.9) | 47 (17.6) | 49 (18.4) | 57 (21.2) |
| Barrier to victim support | 1 (0.4) | 5 (1.9) | 17 (6.4) | 24 (9.0) | 31 (11.6) | 38 (14.2) |

*Note.* *N* = 267. Missing Data Scenario 1: one-third of the sample was randomly selected for missing data generation (*n* = 89). Condition 1 represents the observed data (1.5% missing data rate). Conditions 2 to 6 represent the percentage of items that were randomly deleted for each case in this subset. ODARA = Ontario Domestic Assault Risk Assessment.

**4**

*Missing Data Scenario 2 (Data Deleted for 2/3 of the Sample):*

| ODARA item | Condition 1 (observed) | Condition 2 (1%–10%) | Condition 3  (11%–20%) | Condition 4  (21%–30%) | Condition 5  (31%–40%) | Condition 6  (41%–50%) |
| --- | --- | --- | --- | --- | --- | --- |
| Pre-index domestic assault in police/criminal record | 0 (0.0) | 11 (4.1) | 25 (9.4) | 43 (16.1) | 68 (25.5) | 82 (30.7) |
| Pre-index nondomestic assault in police /criminal record | 2 (0.7) | 21 (7.9) | 27 (10.1) | 48 (18.0) | 72 (27.0) | 81 (30.3) |
| Pre-index custodial sentence of 30 days or more | 1 (0.4) | 13 (4.9) | 24 (9.0) | 40 (15.0) | 46 (17.2) | 60 (22.5) |
| Failure on pre-index conditional release | 0 (0.0) | 12 (4.5) | 25 (9.4) | 44 (16.5) | 65 (24.3) | 75 (28.1) |
| Threat to physically harm or kill at index assault | 3 (1.1) | 19 (7.1) | 39 (14.6) | 64 (24.0) | 94 (35.2) | 106 (39.7) |
| Confinement of the partner at index assault | 2 (0.7) | 10 (3.7) | 24 (9.0) | 48 (18.0) | 72 (27.0) | 88 (33.0) |
| Victim concern about future assaults | 19 (7.1) | 36 (13.5) | 50 (18.7) | 71 (26.6) | 95 (35.6) | 104 (39.0) |
| More than one child altogether | 0 (0.0) | 14 (5.2) | 31 (11.6) | 49 (18.4) | 74 (27.7) | 78 (29.2) |
| Victim’s biological child from a previous partner | 1 (0.4) | 13 (4.9) | 26 (9.7) | 53 (19.9) | 73 (27.3) | 91 (34.1) |
| Pre-index violence against a nondomestic victim | 1 (0.4) | 16 (6.0) | 24 (9.0) | 42 (15.7) | 67 (25.1) | 84 (31.5) |
| Two or more indicators of substance abuse | 1 (0.4) | 19 (7.1) | 28 (10.5) | 46 (17.2) | 65 (24.3) | 89 (33.3) |
| Assault on index victim when pregnant | 22 (8.2) | 32 (12.0) | 47 (17.6) | 72 (27.0) | 78 (29.2) | 89 (33.3) |
| Barrier to victim support | 1 (0.4) | 13 (4.9) | 32 (12.0) | 42 (15.7) | 56 (21.0) | 74 (27.7) |

*Note.* *N* = 267. Missing Data Scenario 2: two-thirds of the sample was randomly selected for missing data generation (*n* = 178). Condition 1 represents the observed data (1.5% missing data rate). Conditions 2 to 6 represent the percentage of items that were randomly deleted for each case in this subset. ODARA = Ontario Domestic Assault Risk Assessment.

**5**

*Missing Data Scenario 3 (Data Deleted for 3/3 of the Sample):*

| ODARA item | Condition 1 (observed) | Condition 2 (1%–10%) | Condition 3  (11%–20%) | Condition 4  (21%–30%) | Condition 5  (31%–40%) | Condition 6  (41%–50%) |
| --- | --- | --- | --- | --- | --- | --- |
| Pre-index domestic assault in police/criminal record | 0 (0.0) | 18 (6.7) | 39 (14.6) | 68 (25.5) | 102 (38.2) | 120 (44.9) |
| Pre-index nondomestic assault in police /criminal record | 2 (0.7) | 20 (7.5) | 37 (13.9) | 74 (27.7) | 95 (35.6) | 116 (43.4) |
| Pre-index custodial sentence of 30 days or more | 1 (0.4) | 24 (9.0) | 35 (13.1) | 50 (18.7) | 68 (25.5) | 91 (34.1) |
| Failure on pre-index conditional release | 0 (0.0) | 20 (7.5) | 36 (13.5) | 68 (25.5) | 98 (36.7) | 123 (46.1) |
| Threat to physically harm or kill at index assault | 3 (1.1) | 26 (9.7) | 55 (20.6) | 98 (36.7) | 118 (44.2) | 137 (51.3) |
| Confinement of the partner at index assault | 2 (0.7) | 18 (6.7) | 44 (16.5) | 76 (28.5) | 112 (41.9) | 130 (48.7) |
| Victim concern about future assaults | 19 (7.1) | 38 (14.2) | 64 (24.0) | 96 (36.0) | 135 (50.6) | 151 (56.6) |
| More than one child altogether | 0 (0.0) | 26 (9.7) | 45 (16.9) | 77 (28.8) | 105 (39.3) | 121 (45.3) |
| Victim’s biological child from a previous partner | 1 (0.4) | 20 (7.5) | 45 (16.9) | 76 (28.5) | 111 (41.6) | 134 (50.2) |
| Pre-index violence against a nondomestic victim | 1 (0.4) | 17 (6.4) | 36 (13.5) | 69 (25.8) | 111 (41.6) | 129 (48.3) |
| Two or more indicators of substance abuse | 1 (0.4) | 22 (8.2) | 40 (15.0) | 69 (25.8) | 103 (38.6) | 143 (53.6) |
| Assault on index victim when pregnant | 22 (8.2) | 42 (15.7) | 58 (21.7) | 82 (30.7) | 115 (43.1) | 130 (48.7) |
| Barrier to victim support | 1 (0.4) | 21 (7.9) | 48 (18.0) | 61 (22.8) | 95 (35.6) | 108 (40.4) |

*Note.* *N* = 267. Missing Data Scenario 3: the entire sample was used for missing data generation. Condition 1 represents the observed data (1.5% missing data rate). Conditions 2 to 6 represent the percentage of items that were randomly deleted for each case in the dataset. ODARA = Ontario

Domestic Assault Risk Assessment.

**6**

*Scale Descriptives for the Observed Data (Distribution of Item Scores and Item Means)*

| SARA-V2 item |  | Item score | |  |
| --- | --- | --- | --- | --- |
|  | 0: *n* (%) | 1: *n* (%) | 2: *n* (%) | *M* (SD) |
| Past assault of family members | 131 (49.1) | 24 (9.0) | 112 (41.9) | 0.93 (0.95) |
| Past assault of strangers or acquaintances | 59 (22.1) | 18 (6.7) | 190 (71.2) | 1.49 (0.83) |
| Past violation of conditional release/supervision | 42 (15.7) | 30 (11.2) | 195 (73.0) | 1.57 (0.75) |
| Recent relationship problems | 15 (5.6) | 27 (10.1) | 225 (84.3) | 1.79 (0.53) |
| Recent employment problems | 76 (28.5) | 67 (25.1) | 124 (46.4) | 1.18 (0.85) |
| Victim of and/or witness to family violence | 142 (53.2) | 52 (19.5) | 73 (27.3) | 0.74 (0.86) |
| Recent substance abuse/dependence | 59 (22.1) | 45 (16.9) | 163 (61.0) | 1.39 (0.83) |
| Recent suicidal or homicidal ideation/intent | 114 (42.7) | 90 (33.7) | 63 (23.6) | 0.81 (0.79) |
| Recent psychotic and/or manic symptoms | 233 (87.3) | 27 (10.1) | 7 (2.6) | 0.15 (0.43) |
| Personality disorder (anger, impulsivity, instability) | 47 (17.6) | 118 (44.2) | 102 (38.2) | 1.21 (0.72) |
| Past physical assault | 12 (4.5) | 6 (2.2) | 249 (93.3) | 1.89 (0.44) |
| Past sexual assault/sexual jealousy | 72 (27.0) | 69 (25.8) | 126 (47.2) | 1.20 (0.84) |
| Past use of weapons and/or credible threats of death | 56 (21.0) | 40 (15.0) | 171 (64.0) | 1.43 (0.82) |
| Recent escalation in frequency or severity of assault | 103 (38.6) | 75 (28.1) | 89 (33.3) | 0.95 (0.85) |
| Past violation of “no contact” orders | 110 (41.2) | 28 (10.5) | 129 (48.3) | 1.07 (0.95) |
| Extreme minimization/denial of spousal assault | 55 (20.6) | 84 (31.5) | 128 (47.9) | 1.27 (0.78) |
| Attitudes that support or condone spousal assault | 47 (17.6) | 115 (43.1) | 105 (39.3) | 1.22 (0.72) |
| Severe and/or sexual assault | 42 (15.7) | 148 (55.4) | 77 (28.8) | 1.13 (0.66) |
| Use of weapons and/or credible threats of death | 110 (41.2) | 32 (12.0) | 125 (46.8) | 1.06 (0.94) |
| Violation of “no contact” order | 181 (67.8) | 12 (4.5) | 74 (27.7) | 0.60 (0.89) |
| Overall distribution of item scores | 1,706 (31.9) | 1,107 (20.7) | 2,527 (47.3) | - |

*Note.* *N* = 267. All cases have complete data. Item scores: 0 = *no/absent*, 1 = *possibly/partially present*, 2 = *yes/present*. SARA-V2 = Spousal Assault

Risk Assessment - Version 2.

**7**

*Missing Data Scenario 1 (Data Deleted for 1/3 of the Sample):*

| SARA-V2 item |  |  | Missing data: *n* (%) | |  |  |
| --- | --- | --- | --- | --- | --- | --- |
|  | Condition 1  (observed) | Condition 2  (1%–10%) | Condition 3 | Condition 4   | Condition 5 | Condition 6 |
| Past assault of family members | 0 (0.0) | 6 (2.2) |  |  |  |  |
| Past assault of strangers or acquaintances | 0 (0.0) | 8 (3.0) | 16 (6.0) | 22 (8.2) | 26 (9.7) | 38 (14.2) |
| Past violation of conditional release/supervision | 0 (0.0) | 6 (2.2) | 10 (3.7) | 15 (5.6) | 29 (10.9) | 30 (11.2) |
| Recent relationship problems | 0 (0.0) | 14 (5.2) | 16 (6.0) | 24 (9.0) | 37 (13.9) | 41 (15.4) |
| Recent employment problems | 0 (0.0) | 7 (2.6) | 17 (6.4) | 31 (11.6) | 43 (16.1) | 59 (22.1) |
| Victim of and/or witness to family violence | 0 (0.0) | 3 (1.1) | 11 (4.1) | 22 (8.2) | 32 (12.0) | 44 (16.5) |
| Recent substance abuse/dependence | 0 (0.0) | 7 (2.6) | 20 (7.5) | 30 (11.2) | 38 (14.2) | 49 (18.4) |
| Recent suicidal or homicidal ideation/intent | 0 (0.0) | 10 (3.7) | 23 (8.6) | 29 (10.9) | 39 (14.6) | 43 (16.1) |
| Recent psychotic and/or manic symptoms | 0 (0.0) | 4 (1.5) | 14 (5.2) | 24 (9.0) | 32 (12.0) | 41 (15.4) |
| Personality disorder (anger, impulsivity, instability) | 0 (0.0) | 5 (1.9) | 12 (4.5) | 18 (6.7) | 29 (10.9) | 41 (15.4) |
| Past physical assault | 0 (0.0) | 11 (4.1) | 22 (8.2) | 29 (10.9) | 33 (12.4) | 47 (17.6) |
| Past sexual assault/sexual jealousy | 0 (0.0) | 8 (3.0) | 17 (6.4) | 21 (7.9) | 28 (10.5) | 30 (11.2) |
| Past use of weapons and/or credible threats of death | 0 (0.0) | 7 (2.6) | 13 (4.9) | 18 (6.7) | 35 (13.1) | 41 (15.4) |
| Recent escalation in frequency or severity of assault | 0 (0.0) | 7 (2.6) | 17 (6.4) | 29 (10.9) | 35 (13.1) | 39 (14.6) |
| Past violation of “no contact” orders | 0 (0.0) | 2 (0.7) | 10 (3.7) | 24 (9.0) | 40 (15.0) | 39 (14.6) |
| Extreme minimization/denial of spousal assault | 0 (0.0) | 6 (2.2) | 16 (6.0) | 24 (9.0) | 30 (11.2) | 44 (16.5) |
| Attitudes that support or condone spousal assault | 0 (0.0) | 8 (3.0) | 19 (7.1) | 26 (9.7) | 37 (13.9) | 46 (17.2) |
| Severe and/or sexual assault | 0 (0.0) | 5 (1.9) | 11 (4.1) | 26 (9.7) | 33 (12.4) | 43 (16.1) |
| Use of weapons and/or credible threats of death | 0 (0.0) | 3 (1.1) | 18 (6.7) | 25 (9.4) | 32 (12.0) | 47 (17.6) |
| Violation of “no contact” order | 0 (0.0) | 4 (1.5) | 16 (6.0) | 31(11.6) | 27 (10.1) | 42 (15.7) |

*Note.* *N* = 267. Missing Data Scenario 1: one-third of the sample was randomly selected for missing data generation (*n* = 89). Condition 1 represents the observed data (no missingness). Conditions 2 to 6 represent the percentage of items that were randomly deleted for each case in this subset.

SARA-V2 = Spousal Assault Risk Assessment - Version 2.

**8**

*Missing Data Scenario 2 (Data Deleted for 2/3 of the Sample):*

| SARA-V2 item |  |  | Missing data: *n* (%) | |  |  |
| --- | --- | --- | --- | --- | --- | --- |
|  | Condition 1  (observed) | Condition 2  (1%–10%) | Condition 3 | Condition 4   | Condition 5 | Condition 6 |
| Past assault of family members | 0 (0.0) | 8 (3.0) |  |  |  |  |
| Past assault of strangers or acquaintances | 0 (0.0) | 16 (6.0) | 30 (11.2) | 53 (19.9) | 64 (24.0) | 75 (28.1) |
| Past violation of conditional release/supervision | 0 (0.0) | 10 (3.7) | 19 (7.1) | 25 (9.4) | 41 (15.4) | 57 (21.3) |
| Recent relationship problems | 0 (0.0) | 11 (4.1) | 35 (13.1) | 48 (18.0) | 70 (26.2) | 89 (33.3) |
| Recent employment problems | 0 (0.0) | 15 (5.6) | 45 (16.9) | 69 (25.8) | 81 (30.3) | 100 (37.5) |
| Victim of and/or witness to family violence | 0 (0.0) | 10 (3.7) | 29 (10.9) | 50 (18.7) | 68 (25.5) | 87 (32.6) |
| Recent substance abuse/dependence | 0 (0.0) | 19 (7.1) | 40 (15.0) | 56 (21.0) | 71 (26.6) | 83 (31.1) |
| Recent suicidal or homicidal ideation/intent | 0 (0.0) | 18 (6.7) | 40 (15.0) | 50 (18.7) | 68 (25.5) | 88 (33.0) |
| Recent psychotic and/or manic symptoms | 0 (0.0) | 12 (4.5) | 36 (13.5) | 55 (20.6) | 76 (28.5) | 86 (32.2) |
| Personality disorder (anger, impulsivity, instability) | 0 (0.0) | 10 (3.7) | 33 (12.4) | 47 (17.6) | 62 (23.2) | 88 (33.0) |
| Past physical assault | 0 (0.0) | 18 (6.7) | 37 (13.9) | 52 (19.5) | 68 (25.5) | 87 (32.6) |
| Past sexual assault/sexual jealousy | 0 (0.0) | 14 (5.2) | 27 (10.1) | 39 (14.6) | 57 (21.3) | 72 (27.0) |
| Past use of weapons and/or credible threats of death | 0 (0.0) | 10 (3.7) | 25 (9.4) | 45 (16.9) | 75 (28.1) | 93 (34.8) |
| Recent escalation in frequency or severity of assault | 0 (0.0) | 16 (6.0) | 32 (12.0) | 48 (18.0) | 63 (23.6) | 80 (30.0) |
| Past violation of “no contact” orders | 0 (0.0) | 8 (3.0) | 26 (9.7) | 43 (16.1) | 67 (25.1) | 70 (26.2) |
| Extreme minimization/denial of spousal assault | 0 (0.0) | 14 (5.2) | 36 (13.5) | 54 (20.2) | 59 (22.1) | 85 (31.8) |
| Attitudes that support or condone spousal assault | 0 (0.0) | 15 (5.6) | 29 (10.9) | 43 (16.1) | 67 (25.1) | 86 (32.2) |
| Severe and/or sexual assault | 0 (0.0) | 10 (3.7) | 27 (10.1) | 49 (18.4) | 63 (23.6) | 99 (37.1) |
| Use of weapons and/or credible threats of death | 0 (0.0) | 14 (5.2) | 25 (9.4) | 47 (17.6) | 82 (30.7) | 92 (34.5) |
| Violation of “no contact” order | 0 (0.0) | 15 (5.6) | 22 (8.2) | 54 (20.2) | 69 (25.7) | 85 (31.8) |

*Note.* *N* = 267. Missing Data Scenario 2: two-thirds of the sample was randomly selected for missing data generation (*n* = 178). Condition 1 represents the observed data (no missingness). Conditions 2 to 6 represent the percentage of items that were randomly deleted for each case in this subset. SARA-V2 = Spousal Assault Risk Assessment - Version 2.

**9**

*Missing Data Scenario 3 (Data Deleted for 3/3 of the Sample):*

| SARA-V2 item |  |  | Missing data: *n* (%) | |  |  |
| --- | --- | --- | --- | --- | --- | --- |
|  | Condition 1  (observed) | Condition 2  (1%–10%) | Condition 3 | Condition 4   | Condition 5 | Condition 6 |
| Past assault of family members | 0 (0.0) | 13 (4.9) |  |  |  |  |
| Past assault of strangers or acquaintances | 0 (0.0) | 17 (6.4) | 50 (18.7) | 65 (24.3) | 94 (35.2) | 115 (43.1) |
| Past violation of conditional release/supervision | 0 (0.0) | 14 (5.2) | 25 (9.4) | 43 (16.1) | 84 (31.5) | 108 (40.4) |
| Recent relationship problems | 0 (0.0) | 19 (7.1) | 45 (16.9) | 76 (28.5) | 107 (40.1) | 130 (48.7) |
| Recent employment problems | 0 (0.0) | 26 (9.7) | 64 (24.0) | 87 (32.6) | 109 (40.8) | 137 (51.3) |
| Victim of and/or witness to family violence | 0 (0.0) | 19 (7.1) | 48 (18.0) | 79 (29.6) | 99 (37.1) | 120 (44.9) |
| Recent substance abuse/dependence | 0 (0.0) | 26 (9.7) | 56 (21.0) | 84 (31.5) | 100 (37.5) | 146 (54.7) |
| Recent suicidal or homicidal ideation/intent | 0 (0.0) | 24 (9.0) | 54 (20.2) | 83 (31.1) | 101 (37.8) | 130 (48.7) |
| Recent psychotic and/or manic symptoms | 0 (0.0) | 21 (7.9) | 58 (21.7) | 82 (30.7) | 105 (39.3) | 125 (46.8) |
| Personality disorder (anger, impulsivity, instability) | 0 (0.0) | 19 (7.1) | 42 (15.7) | 75 (28.1) | 100 (37.5) | 125 (46.8) |
| Past physical assault | 0 (0.0) | 20 (7.5) | 46 (17.2) | 74 (27.7) | 100 (37.5) | 136 (50.9) |
| Past sexual assault/sexual jealousy | 0 (0.0) | 17 (6.4) | 40 (15.0) | 65 (24.3) | 95 (35.6) | 116 (43.4) |
| Past use of weapons and/or credible threats of death | 0 (0.0) | 13 (4.9) | 49 (18.4) | 81 (30.3) | 109 (40.8) | 122 (45.7) |
| Recent escalation in frequency or severity of assault | 0 (0.0) | 27 (10.1) | 44 (16.5) | 76 (28.5) | 98 (36.7) | 128 (47.9) |
| Past violation of “no contact” orders | 0 (0.0) | 17 (6.4) | 36 (13.5) | 67 (25.1) | 94 (35.2) | 113 (42.3) |
| Extreme minimization/denial of spousal assault | 0 (0.0) | 21 (7.9) | 55 (20.6) | 72 (27.0) | 108 (40.4) | 140 (52.4) |
| Attitudes that support or condone spousal assault | 0 (0.0) | 21 (7.9) | 42 (15.7) | 69 (25.8) | 104 (39.0) | 119 (44.6) |
| Severe and/or sexual assault | 0 (0.0) | 20 (7.5) | 43 (16.1) | 79 (29.6) | 99 (37.1) | 129 (48.3) |
| Use of weapons and/or credible threats of death | 0 (0.0) | 21 (7.9) | 34 (12.7) | 66 (24.7) | 98 (36.7) | 141 (52.8) |
| Violation of “no contact” order | 0 (0.0) | 16 (6.0) | 49 (18.4) | 62 (23.2) | 92 (34.5) | 123 (46.1) |

*Note.* *N* = 267. Missing Data Scenario 3: the entire sample was used for missing data generation. Condition 1 represents the observed data (no missingness). Conditions 2 to 6 represent the percentage of items that were randomly deleted for each case in the dataset. SARA-V2 = Spousal Assault Risk Assessment - Version 2.
